# Lateralization of Sucrose Responsiveness and Non-associative Learning in Honeybees

**DOI:** 10.3389/fpsyg.2018.00425

**Published:** 2018-03-28

**Authors:** David Baracchi, Elisa Rigosi, Gabriela de Brito Sanchez, Martin Giurfa

**Affiliations:** ^1^Centre de Recherches sur la Cognition Animale, Centre de Biologie Intégrative (CBI), Université Toulouse III Paul Sabatier, Toulouse, France; ^2^Centre National de la Recherche Scientifique, Université Paul Sabatier, Toulouse, France; ^3^Department of Biology, Lund University, Lund, Sweden

**Keywords:** *Apis mellifera*, behavioral lateralization, brain asymmetries, habituation, left–right asymmetries, proboscis extension response, sucrose sensitivity

## Abstract

Lateralization is a fundamental property of the human brain that affects perceptual, motor, and cognitive processes. It is now acknowledged that left–right laterality is widespread across vertebrates and even some invertebrates such as fruit flies and bees. Honeybees, which learn to associate an odorant (the conditioned stimulus, CS) with sucrose solution (the unconditioned stimulus, US), recall this association better when trained using their right antenna than they do when using their left antenna. Correspondingly, olfactory sensilla are more abundant on the right antenna and odor encoding by projection neurons of the right antennal lobe results in better odor differentiation than those of the left one. Thus, lateralization arises from asymmetries both in the peripheral and central olfactory system, responsible for detecting the CS. Here, we focused on the US component and studied if lateralization exists in the gustatory system of *Apis mellifera*. We investigated whether sucrose sensitivity is lateralized both at the level of the antennae and the fore-tarsi in two independent groups of bees. Sucrose sensitivity was assessed by presenting bees with a series of increasing concentrations of sucrose solution delivered either to the left or the right antenna/tarsus and measuring the proboscis extension response to these stimuli. Bees experienced two series of stimulations, one on the left and the other on the right antenna/tarsus. We found that tarsal responsiveness was similar on both sides and that the order of testing affects sucrose responsiveness. On the contrary, antennal responsiveness to sucrose was higher on the right than on the left side, and this effect was independent of the order of antennal stimulation. Given this asymmetry, we also investigated antennal lateralization of habituation to sucrose. We found that the right antenna was more resistant to habituation, which is consistent with its higher sucrose sensitivity. Our results reveal that the gustatory system presents a peripheral lateralization that affects stimulus detection and non-associative learning. Contrary to the olfactory system, which is organized in two distinct brain hemispheres, gustatory receptor neurons converge into a single central region termed the subesophagic zone (SEZ). Whether the SEZ presents lateralized gustatory processing remains to be determined.

## Introduction

Lateralization, once considered a hallmark of humans (Corballis, 1989), is a rather widespread animal phenomenon [recently reviewed in Rogers et al. (2013) and Rogers and Vallortigara (2017)]. Sensory and motor asymmetries in behavior, as well as asymmetries in the nervous system, occur in many taxa, independently of brain size or complexity (Frasnelli et al., 2012; Rogers et al., 2013; Frasnelli, 2017). It has been suggested that left–right asymmetries avoid duplicate processing of information, optimizing the computation of the nervous system and reducing the possibility of conflicting information from bilateral sensory organs (Vallortigara and Rogers, 2005). Lateralization might also be advantageous at the periphery of sensory systems, as shown by the example of nematodes where functional asymmetries of chemosensory neurons optimize chemotaxis (Suzuki et al., 2008) and are required for odor discrimination (Wes and Bargmann, 2001).

In the last decade, many studies reported the occurrence of sensory asymmetries in various invertebrate species (reviewed in Frasnelli et al., 2012; Frasnelli, 2017). Among insects, the honeybee has received particular attention in the study of asymmetries (reviewed in Frasnelli et al., 2014). Honeybee workers trained to associate either a visual or an olfactory stimulus (the conditioned stimulus, CS) with sugar reward (the unconditioned stimulus, US) show population-level asymmetries in recalling the sensory stimulus (Letzkus et al., 2006, 2008; Rogers and Vallortigara, 2008; Anfora et al., 2010; Frasnelli et al., 2010a,b; Rigosi et al., 2011; Guo et al., 2016). Specifically, bees in which only one antenna/eye is stimulated by the CS show a dominance of the right side in the ability to recall learned sensory stimuli (Letzkus et al., 2006, 2008; Anfora et al., 2010; Frasnelli et al., 2010a; Rigosi et al., 2011), as do bees trained with both antennae in use and tested for short-term memory (Rogers and Vallortigara, 2008; Frasnelli et al., 2010b). A left-side dominance has been reported for the recall of long-term olfactory memory when bees are trained with both antennae in use (Rogers and Vallortigara, 2008; Frasnelli et al., 2010b). In the case of the olfactory system, population-level asymmetries are already present at the level of the antennae and the antennal lobes, with the right side showing a higher number of antennal olfactory sensilla and a higher separation between odors when antennal lobe responses are evaluated using calcium imaging (Letzkus et al., 2006; Frasnelli et al., 2010a; Rigosi et al., 2015). Also, an increased protein-coding gene expression is observed 24 h after olfactory learning in bees trained with their right antennae as compared with bees trained with only the left ones (Guo et al., 2016).

Although lateralization of olfactory processing might be sufficient *per se* to trigger the lateralized behavior in the framework of olfactory learning and memory, the contribution of the US, i.e., the sucrose reward, has been largely overlooked. This is particularly surprising as sucrose perception in olfactory conditioning starts at the level of bilateral organs such as the antennae (Esslen and Kaissling, 1976; de Brito Sanchez et al., 2005) and the tarsi (de Brito Sanchez et al., 2014). It has been shown that food rewards trigger an asymmetrical expression of the immediate early-gene c-jun transcript in the honeybee (McNeill and Robinson, 2015; McNeill et al., 2016). Additionally, sucrose responsiveness affects learning and odor discrimination performance in worker honeybees (Scheiner et al., 2001, 2003), so that any left–right bias in sucrose processing might indeed contribute to the observed behavioral asymmetries in olfactory learning and memory in this insect.

Here, we investigated lateralization of sucrose sensitivity of honeybees, both at the level of the antennae and the tarsi. Using the appetitive response of proboscis extension response (PER) of bees to sucrose solutions of increasing concentrations, we compared sucrose responsiveness at the level of left vs. right antennae and tarsi. We report the first evidence for lateralization of sugar sensitivity in the honeybee, with higher responsiveness and resistance to habituation on the right antenna compared to the left one. We discuss the consequences of this lateralization and propose further research avenues in the study of gustatory lateralization in bees.

## Materials and Methods

### Animal Preparation

**FIGURE 1:**
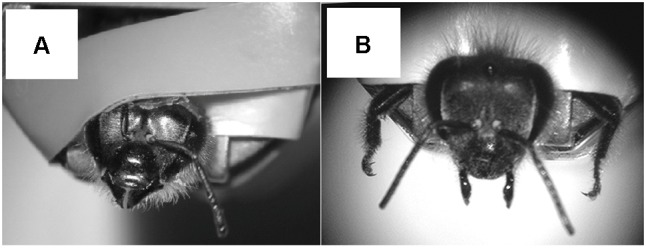
**(A)** A harnessed honeybee prepared for the antennal responsiveness assay with the upper part of the right antenna (flagellum) blocked by a strip of tape (the tape in contact with the antenna is not sticky) in order to prevent any movement and stimulus detection by this antenna during testing. The left antenna is free to move and can be easily reached by the experimenter to test its sucrose sensitivity. **(B)** A harnessed honeybee prepared for the tarsal responsiveness assay with its fore-legs fixed wide open in order to allow tarsal gustatory stimulation.

Experiments were carried on using forager bees (*Apis mellifera ligustica*) caught at a feeder made available each morning at the experimental apiary of the Research Center on Animal Cognition, located in the campus of the University Paul Sabatier (Toulouse, France). Each experimental day, bees were brought to the laboratory, cold anesthetized until immobility (approximately 4–5 min), and harnessed individually within a metal tube using adhesive tape placed in between the head and the thorax. Low-temperature melting wax was used to further immobilize the head (Matsumoto et al., 2012). Bees used in the antennal-responsiveness assay were prepared as follow: two thin strips of adhesive tape (∼4 mm × 5 cm) were joined together on their sticky side and applied to one of the two antennae as shown in **Figure 1A**. This allowed to block one antenna without damaging it during the first experimental phase in which sucrose responsiveness via the contralateral antenna was recorded, and to free it in a second experimental phase to assess sucrose responsiveness while the contralateral antenna was blocked in the same way. PER can be elicited in bees immobilized in this way by gently touching the free antenna with a toothpick soaked with sucrose solution. Bees used for tarsal responsiveness were mounted in the metal tubes with fore-tarsi protruded and fixed wide open in order to facilitate their stimulation (**Figure 1B**). PER can be elicited in these bees by touching the left or the right fore-tarsus with a toothpick soaked with sucrose solution (de Brito Sanchez et al., 2014). Once harnessed, each bee was checked for intact PER and was fed with 5 μl of sucrose solution (50% w/w) to equalize the level of hunger across individuals. After feeding, bees were kept resting for 2 h in a dark and humid place (∼60%) at 25 ± 1°C before proceeding with the experiment. Bees that did not show the reflex were discarded.

### Sucrose Responsiveness Assay

Two hours after resting, sucrose responsiveness was quantified by recording PER in response to increasing concentrations of sucrose, following a standard protocol (Pankiw and Page, 1999; Scheiner et al., 1999; Scheiner et al., 2003). Sucrose solutions were prepared using sucrose of analytical grade (Sigma-Aldrich, France) diluted in purified water (Milli-Q System, Millipore, Bedford, MA, United States). Each bee was presented with seven sucrose solutions of increasing concentrations: 0.1, 0.3, 1, 3, 10, 30, and 50 (w/w), which were delivered on the free antenna or tarsus with the help of one toothpick. In the case of tarsal stimulation, the tarsus was approached from below to avoid any accidental contact with the antennae and care was taken to ensure that the toothpick contacted both the tarsus and the claws. In the case of antennal stimulation, the antenna was approached from below to minimize the interference with the visual system of the insect and the antenna touched on its mid-distal part including the tip. In both cases, successive sucrose stimulations were interspersed with purified water stimulations to avoid sensitization. The inter-stimulus interval (either for sucrose or water) was ∼2 min. Bees that did not respond to any sucrose concentration, including the 50%, were excluded from successive analyses (Scheiner et al., 1999). We also discarded bees responding to water to control for the effect of thirst on sucrose responsiveness and those exhibiting inconsistent responses to sucrose (i.e., responding to a lower but not to a higher sucrose concentration) as preconized by the standard method of sucrose responsiveness evaluation (Pankiw and Page, 1999; Scheiner et al., 1999, 2003). To test for lateralization in sucrose responsiveness, the PER assessment to all sucrose concentrations was repeated twice for each bee, one on each side (left vs. right). To balance out the possible effect of testing order, half of the bees were first stimulated with sucrose on their right antenna/tarsus and then on their left antenna/tarsus, while the other half was subjected to the inversed sequence.

The two sequences of stimulation were spaced by 2 h. In the case of bees tested for antennal responsiveness, the tape covering the antenna was moved to the other antenna soon after the end of the first assay. Bees were then fed again with 5 μl of sucrose solution, kept resting for 2 h in a dark and humid place (∼60%) at 25 ± 1°C before proceeding with the second phase of the experiment. For each bee retained for the analysis (antennal sensitivity: *n* = 101, tarsal sensitivity: *n* = 88), an individual sucrose response score (SRS) was calculated as the number of sucrose concentrations eliciting a PER (e.g., SRS = 4 for an individual responding to 3, 10, 30, and 50% sucrose solution but not to lower concentrations). SRS ranged from 1 (bees responding only to the 50% sucrose solution delivered at the end of the sequence) to 7 (bees responding to all seven concentrations). For each bee two different SRSs were calculated, one for the left antenna/tarsus and one for the right antenna/tarsus.

### Non-associative Learning Assay

A subset of bees tested for left–right antennal lateralization (*n* = 57) was then trained following a habituation protocol to investigate the possible existence of lateralization in habituation to antennal sucrose stimulation. These bees were the last ones tested in the sucrose responsiveness assay. At the end of the previous assay, bees were fed *ad libitum* and kept resting overnight in a dark and humid place (∼60%) at 25 ± 1°C with both antennae free to move. The day after, bees were fed again with 5 μl of sucrose solution (50% w/w) and one antenna was blocked as explained above. After 2 h resting, bees were subjected to the habituation assay, which consisted of 30 successive stimulations with 10% sucrose solution on the free antenna. Stimulations lasted less than a second and the inter-stimulus interval was 10 s (Scheiner, 2004; Baracchi et al., 2017). Once the first habituation phase was finished, the bees had a resting period of 2 h. The habituated antenna was blocked and the non-habituated one was released to perform the second habituation phase in the same way. The same right–left or left–right order was used in the sucrose responsiveness and habituation assays so that if a bee was first tested for sucrose responsiveness on the right antenna and then on left antenna (Right 1 and Left 2, *n* = 27), it was first habituated on the right antenna and then on the left antenna and *vice versa* (Left 1 and Right 2) (*n* = 30).

At the end of each habituation phase, a dishabituation trial (DT) was performed 10 s after the last habituation trial. It consisted of a single stimulation with a 50% sucrose solution delivered to the selected antenna. Ten seconds after the DT, the bees received a test stimulation on the same antenna with the original stimulus used in the habituation phase (10% sucrose solution). In all cases, PER (yes/no) to the stimulating solution was assessed. For each antenna, an individual habituation score (HS) was calculated as the number of sucrose stimulations eliciting a PER in the habituation phase. HSs ranged, therefore, from 1 to 30.

### Data Analysis

Proboscis extension responses (1 or 0) to sucrose stimulation of individual bees in both the sucrose responsiveness and the habituation assays were examined using generalized linear mixed models (GLMMs) with a binomial error structure – logit-link function – *glmer* function of R package *lme4* (Bates et al., 2014). For the sucrose responsiveness assay, either for the tarsal or the antennal experiment, “*Response*” was entered as a dependent variable, “*Side*” and “*Order*” were entered as fixed factors, and “*Sucrose concentration*” was entered as a covariate. For the habituation assay, “*Response*” was the dependent variable, ‘*Order*’ was a fixed factor, and ‘*Trial*’ was entered as a covariate.

Left–right differences in SRS, either at the level of the tarsi and the antennae, were analyzed with a linear mixed model (LMM). The “*SRS*” was the dependent variable, the “*Side*” and the “*Order*” were fixed factors. Left–right differences in antennal HSs were analyzed with a LMM where the “*HS*” was entered as a dependent variable, “*Side*”, “*Order*,” and “*SRS*” were entered as fixed factors. In all models, “*Individual*” identity (ID) was considered as a random factor in order to allow for repeated measurements. In all cases, we retained the significant model with the highest explanatory power (i.e., the lowest AIC value). The interaction *Side* ^∗^ *Order* was evaluated in all the full models but was not significant in all cases and was, therefore, not included in the selected models. Left–right differences in the response to the DT were tested with a Wilcoxon test, while dishabituation responses to the original stimulus used for habituation were tested with χ^2^ test. All statistical analyses were performed with R 3.2.3 (R Core Team, 2016).

## Results

**FIGURE 2:**
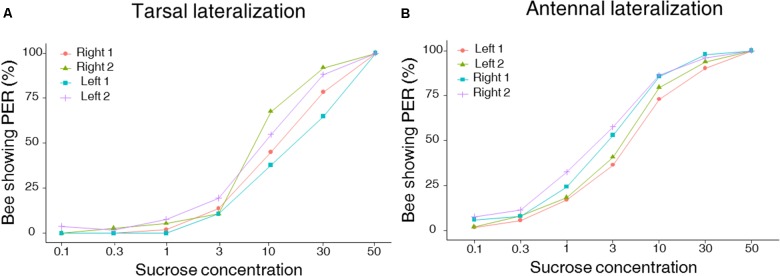
Left–right tarsal (fore-tarsi) **(A)** and antennal **(B)** responsiveness to sucrose solution. Both graphs show cumulative percentages of bees showing PER when stimulated with seven sucrose solutions of increasing concentration (0.1, 0.3, 1, 3, 10, 30, and 50% w/w). Approximately half of the bees were tested first on the right antenna (Right 1) and then on the left antenna (Left 2) (*n* = 49) and *vice versa* (Left 1 and Right 2) (*n* = 52). Similarly, about half of the bees were tested first on the right tarsus (Right 1) and then on the left tarsus (Left 2) (*n* = 51) and *vice versa* (Left 1 and Right 2) (*n* = 37). **(A)** No lateralization of tarsal sucrose sensitivity was found (GLMM, *p* = 0.10), but a significant effect of the stimulation sequence was detected (GLMM, *p* = 0.014), with sensitivity being increased during the second stimulation phase. **(B)** A lateralization of antennal sucrose sensitivity was found at the population level, with the right antenna being significantly more sensitive to sucrose than the left one (GLMM, *p* < 0.0001).

**FIGURE 3:**
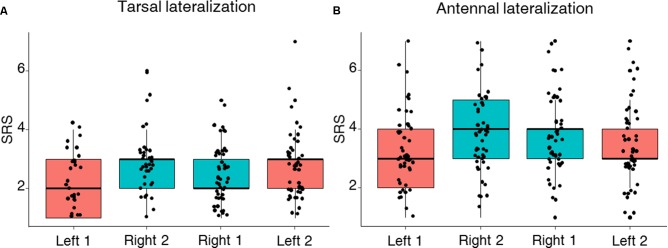
Left–right tarsal (fore-tarsi) **(A)** and antennal **(B)** individual sucrose response scores (SRS). Median, quartiles, and max and min (upper and lower whiskers) SRS values of bees stimulated with seven sucrose solutions of increasing concentration (from 0.1 to 50% w/w) on the left (reddish) and the right (cyan) antenna and tarsus. Black dots represent individual bees. For each bee, SRS values could range between 7 (a bee responding to all seven concentrations) and 1 (a bee responding only to the highest concentration of 50%). **(A)** No lateralization of sucrose sensitivity was found at the level of the tarsi (LMM, *n* = 88, *p* = 0.29) while the order of testing had a significant effect (LMM, *p* = 0.001). **(B)** SRS revealed a lateralization of sucrose sensitivity (LMM, *n* = 101, *p* = 0.0001) while the order of testing had no effect (LMM, *p* = 0.17).

Using the PER to sucrose solutions of increasing concentration, we investigated lateralization of sucrose sensitivity at the level of the antennae and the tarsi in two independent groups of bees. As expected, PER of harnessed bees increased with sucrose concentrations in both the group of bees tested on the antennae and the one tested on the tarsi of the fore-legs (**Figure 2**; GLMM, *Sucrose concentration:* antennae: χ^2^ = 153.4, *df* = 1, *n* = 101, *p* < 0.0001; tarsi: χ^2^ = 241.8, *df* = 1, *n* = 88, *p* < 0.0001). Yet, differences in the patterns of responsiveness were found when comparing antennal and tarsal sucrose responsiveness. Bees tested on the tarsi showed the same level of responsiveness on both sides (GLMM, *Side*: χ^2^ = 2.69, *df* = 1, *n* = 88, *p* < 0.10) but the order of testing affected sucrose responsiveness: in the second stimulation phase, bees responded significantly more, irrespectively of the tarsal side considered (GLMM, *Order*: χ^2^ = 6.04, *df* = 1, *n* = 88, *p* = 0.014, **Figure 2A**). Accordingly, the tarsal SRS, which provides an individual assessment of sucrose responsiveness, was statistically similar on the left and the right side (mean SRS ± SEM: mean right 1 and 2 side 2.6 ± 0.06; mean left 1 and 2 side 2.5 ± 0.07; LMM, χ^2^ = 1.08, *df* = 1, *n* = 88, *p* = 0.29) but differed between stimulation phases (**Figure 3A**; LMM, χ^2^ = 10.47, *n* = 88, *p* = 0.001).

In the case of bees tested on the antennae, we found a lateralization of sucrose sensitivity when comparing the left and right sides (**Figure 2B**; GLMM, *Side*: χ^2^ = 19.30, *df* = 1, *n* = 101, *p* < 0.0001). In particular, the right antenna was more sensitive to sucrose than the left one. This was particularly visible for intermediate sucrose concentrations such as 3% but not for the highest concentration where all groups showed maximal responsiveness (left–right: χ^2^ test: 0.1%: *p* = 0.09; 0.3%: *p* = 0.32; 1%: *p* = 0.07; 3%: *p* = 0.02; 10%: *p* = 0.07; 30%: *p* = 0.12), irrespectively of the stimulation phase considered (GLMM, *Order*: χ^2^ = 2.71, *df* = 1, *n* = 101, *p* = 0.1). The SRS analysis confirmed the antennal lateralization detected at the population level (**Figure 3B**; mean SRS ± SEM: right 1 and 2 side 3.9 ± 0.08; left 1 and 2 side 3.3 ± 0.08; LMM, χ^2^ = 14.70, *n* = 101, *p* = 0.00012) and that the order of testing had no effect (LMM, χ^2^ = 1.86, *p* = 0.17). Thus, while the fore-tarsi did not show evidence of lateralization, the antennae showed a clear asymmetry in sucrose responsiveness.

**FIGURE 4:**
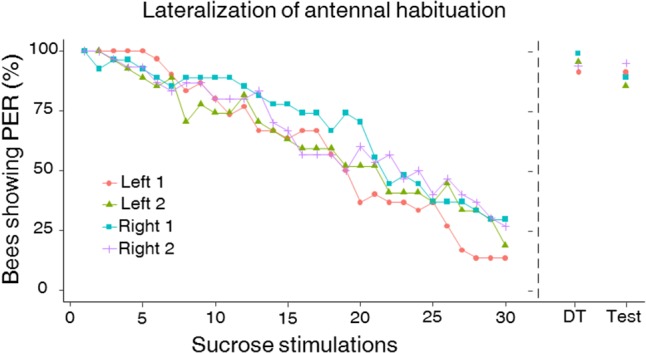
Left–right antennal habituation to sucrose solution stimulation. Like in the sucrose responsiveness assays, approximately half of the bees were first tested for habituation on the right antenna (Right 1) and then on the left antenna (Left 2) (*n* = 27) while the other half experienced the reversed sequence (Left 1 and Right 2; *n* = 30). The two sequences of side stimulation were spaced by 2 h. Habituation consisted in 30 consecutive stimulations with a 10% (w/w) sucrose solution on the free antenna (while the other one was blocked). Ten seconds after the last habituation trial, bees were stimulated on the habituated antenna with a 50% (w/w) sucrose stimulation to induce dishabituation (“dishabituation trial” or DT). Ten seconds after the DT, bees were stimulated on the same antenna with the original stimulus used during the training (i.e., 10% sucrose solution) to check for typical response recovery following dishabituation (“Test”). The right antenna was more resistant to habituation than the left one at the population level (GLMM, *p* = 0.006). Habituation to sucrose stimulation was significantly affected by the SRS of individual bees (GLMM, *p* < 0.0001), thus demonstrating that the left–right antennal asymmetry in sucrose sensitivity translates directly into a lateralization of habituation to sucrose stimulation. The order of testing had no effect on habituation. No significant left–right differences in the DT as well as in the test were observed (Wilcoxon test, DT: *p* = 0.41, test: *p* = 0.36). The DT as well as re-stimulating with the original stimulus induced a significant response recovery, which did not differ between sides. This demonstrates that the observed decrease in PER to the 10% sucrose solution was a real case of habituation and was not due to sensory adaptation or fatigue.

**FIGURE 5:**
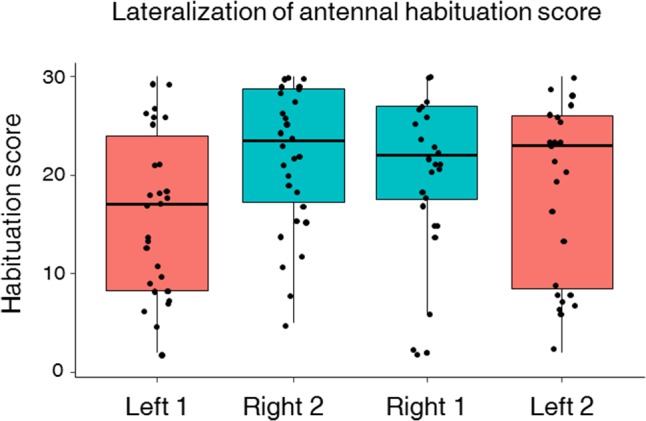
Left–right antennal habituation score (HS). Median, quartiles, and max and min (upper and lower whiskers) sucrose response values of individual HSs for bees subjected to two phases of 30 consecutive antennal stimulations with 10% sucrose solution. Approximately half of the bees were tested for habituation first on the right antenna (Right 1) and then on the left antenna (Left 2) (*n* = 27) and *vice versa* (Left 1 and Right 2) (*n* = 30). Black dots represent individual bees. Bees with a score of 30 responded to all 30 sucrose stimulations, i.e., did not show any habituation. The right antenna had a higher score than the left antenna (LMM, *p* = 0.001) indicating a higher resistance to habituation on the right side compared to the left one. The order of testing had no significant effect on the HS.

This asymmetry led us to investigate antennal lateralization of habituation to antennal sucrose stimulation in a subset of bees previously tested for left–right antennal lateralization (*n* = 57). Bees were first habituated on one antenna and afterward on the other antenna. In both phases, the repeated stimulation with 10% sucrose solution led to significant habituation along trials as PER decreased significantly from the 1st to the 30th habituation trial (**Figure 4**; GLMM, *trial*: χ^2^ = 683.2, *df* = 1, *p* < 0.0001). Yet, the degree of PER habituation differed between the left and the right antenna (**Figure 4**; GLMM, *Side*: χ^2^ = 29.78, *df* = 1, *p* < 0.0001) and was independent of the order of testing (GLMM, *Order*: χ^2^ = 0.33, *df* = 1, *p* = 0.56). Consistently with the higher sensitivity of the right antenna found in the prior experiment, we found that the right antenna was also more resistant to habituation than the left one at the population level, irrespectively of the order of testing. Accordingly, a higher HS was found for the right antenna (i.e., less habituation) compared to the left antenna (**Figure 5**; mean HS ± SEM: right 1 and 2 side, 21.4 ± 0.56; left 1 and 2 side, 17.5 ± 0.64; LMM, χ^2^ = 11.30, *df* = 1, *p* = 0.001). The SRS of each antenna had indeed a main effect on its HS, thus showing that higher sucrose sensitivity resulted in more resistance to habituation (LMM, χ^2^ = 33.86, *df* = 1, *p* < 0.0001).

The recovery of PER after replacing the 10% habituation sucrose solution by a 50% sucrose solution (**Figure 4**; dishabituation trial, DT) ruled out that the observed decrease of PER to successive stimulations was due to fatigue and/or sensory adaptation. Indeed, a significant increase of PER was observed between the response in the last habituation trial and in the DT in all cases (**Figure 4**; Wilcoxon test, *left 1*: *n* = 30, *Z* = -4.79, *p* < 0.001; *left 2*: *n* = 27, *Z* = -4.58, *p* < 0.001; *right 1*: *n* = 27, *Z* = -4.36, *p* < 0.001; *right 2*: *n* = 30, *Z* = -4.47, *p* < 0.001). Stimulating with the original habituating stimulus (10% sucrose solution) in the final test after the DT showed response recovery following dishabituation; in all cases, responses recorded in this last test were significantly higher than those recorded in the last habituation trial (**Figure 4**; Wilcoxon test, all groups *p* < 0.001). Significant left–right differences were neither found in dishabituation nor in the final test (Wilcoxon test, DT: *Z* = -0.81, *p* = 0.41; test: *Z* = -0.90, *p* = 0.36).

## Discussion

In the present work, we studied for the first time gustatory lateralization in the honeybee by testing their sucrose sensitivity both at the level of the antennae and the distal segments of the fore-legs (tarsi). We found that a left–right asymmetry in sucrose sensitivity exists at the level of the antennae. Bees exhibited a higher responsiveness to intermediate sucrose concentrations on the right than on the left antenna, an effect that was independent of the order of stimulation. This asymmetry was also visible in a habituation experiment, where repeated stimulation with an intermediate sucrose concentration on the more sensitive right antenna determined less habituation than on the left antenna. No lateralization was found at the level of the fore-tarsi where, on the contrary, enhanced responsiveness was found on the second phase of sucrose stimulation, irrespectively of tarsal side. In this case, the successive experience with sucrose seemed to enhance the sensitivity of the bees for both tarsi.

Recall of olfactory memory is lateralized in honeybees as they achieve better retention performances when the odorant acting as CS is delivered to their right rather than to their left antenna after olfactory PER conditioning with single antenna in use (Frasnelli et al., 2014; Guo et al., 2016). Interestingly, when the odorant is delivered to both antennae at the same time during training and presented to single antennae during test, the memory recall is achieved better with the right antenna only 1–2 h after training, while at 6–23 h the recall is better performed with the left antenna (Rogers and Vallortigara, 2008). The mechanisms underlying this asymmetry remain to be clarified but, most likely, they can be partially retraced to left–right differences both at the peripheral and at the central level. At the peripheral level, olfactory sensilla (i.e., *sensilla placodea*, *trichodea*, and *basiconica*) have been found to be more abundant on the right than on the left antenna, suggesting that lateralization in memory retrieval may arise from asymmetries in the detection of the conditioned odor stimulus (CS) during appetitive olfactory training (Letzkus et al., 2006; Frasnelli et al., 2010a). At the central level, neural responses in the left and right antennal lobes differ, so that odor encoding in these structures results in higher separation (i.e., better discriminability) in the right antennal lobe (Rigosi et al., 2015). Moreover, 24 h after olfactory PER conditioning with single antennae, the right side of the brain shows increased gene-expression compared to the left one (Guo et al., 2016).

These findings clearly underline that asymmetries in olfactory retrieval have a correlate at various levels of odorant (CS) processing in the bee nervous system. However, these asymmetries might also correlate with additional asymmetries at the level of gustatory (US) processing. In olfactory PER conditioning, sucrose is the US used to induce PER. In the first versions of this protocol, sucrose was delivered to the fore-tarsi and then to the proboscis (Takeda, 1961) while in the more recent and standard protocol, it is first delivered to the antennae and then to the proboscis (Matsumoto et al., 2012). Among these gustatory appendages, only the antennae exhibited a differential sucrose sensitivity between the left and the right side. However, it worth noting that this asymmetry was only visible for sucrose concentrations that are typically not used in olfactory PER condition as they are too low (e.g., 3%) to support efficient learning (Matsumoto et al., 2012). The same remark may apply to other conditioning protocols. For instance, in a recently established gustatory conditioning protocol, bees receive tastants on the antennae and, afterward, a mild electric shock which induces the sting extension reflex (SER) (Guiraud et al., 2018). Over the successive trials, bees learn to extend the sting to aversive tastes. No left–right antennal asymmetries were found when bees learned the association between sucrose and the shock. Yet, the concentration of sucrose (33%) used in this protocol (Guiraud et al., 2018) falls within the range in which asymmetries were no longer evident in our work. Interestingly, contrary to olfactory PER conditioning, in gustatory SER conditioning, sucrose can be used at concentrations lower than 33% as it does not represent the US but the CS (Guiraud et al., 2018). As sucrose sensitivity is a crucial determinant of learning performance in an associative learning task, we predict that the antennal asymmetry in sucrose sensitivity revealed by our work is likely to translate into an asymmetrical performance in aversive gustatory learning and/or memory formation when low sucrose concentrations are used as CS.

Asymmetric performances during olfactory learning between bees with only their left or their right antenna in use have been reported only for learning to detect an odorant from a background but not during differential odor learning (Rigosi et al., 2015). Interestingly, we demonstrated that a left–right asymmetry exists in a simple form of non-associative learning (habituation). As expected, the observed lateralization in sucrose responsiveness (i.e., higher sucrose sensitivity on the right antenna) corresponded to a lateralization in the same direction in habituation to successive sucrose stimulations (i.e., more resistance to habituation upon stimulation on the right antenna). This finding is not surprising given the correlation existing between sucrose responsiveness and habituation to antennal sucrose stimulation in bees (Scheiner, 2004).

The mechanisms underlying left–right sucrose responsiveness asymmetries in the antennae may also involve left–right asymmetries at the peripheral level, i.e., in the gustatory sensilla/receptors located on these appendages. Analyses of non-olfactory sensilla located on the bee antennae, which included a category of gustatory sensilla (i.e., *sensilla chaetica*), found slightly more sensilla in the segments 3rd–9th of the left than on the right antenna (Frasnelli et al., 2010a). This asymmetry does not align with our finding that bees are more sensitive to sucrose on the right antenna. However, when the last distal segment of the flagellum (10th segment) which constitutes the primary antennal contact region was considered, the situation was reversed with slightly more non-olfactory sensilla on the right than on the left antenna (Frasnelli et al., 2010a). Importantly, *sensilla chaetica*, which are responsible for sucrose detection and respond in a dose-dependent manner to sucrose solution (Haupt, 2004; de Brito Sanchez et al., 2005), show a high degree of variability in spike frequency and sucrose response within the same antenna (Haupt, 2004). Lateralization of sucrose responsiveness might be, therefore, due to left–right differences in the proportion of distal *sensilla chaetica* with different sensitivities to sucrose rather than to differences in their absolute number.

Besides the antennae, other body regions such as the subesophagic zone (SEZ) of the brain might be involved in the observed left–right lateralization. The SEZ is the main central gustatory area in the insect brain (see de Brito Sanchez, 2011). It has a major role in gustatory encoding but also participates in the motor control of mouthparts and mechanosensory information processing. Contrary to the olfactory system, which is organized in two distinct brain hemispheres, the SEZ is a central unpaired brain area. Several sucrose processing neurons with their soma located in the ventral and median region of the SEZ (VUM neurons: ventral unpaired median neurons) and arborizing within different regions of the bee brain have been reported (Schröter et al., 2006). Whether, despite its unpaired nature, the SEZ presents a lateralized gustatory processing contributing to the observed antennal gustatory asymmetries remains to be determined. Interestingly, gustatory receptor neurons hosted by antennal gustatory sensilla do not only project to the unpaired SEZ (Pareto, 1972; Suzuki, 1975; Haupt, 2007) but also to two adjacent lateral regions termed the lateral lobes (one on each side of the SEZ) (Haupt, 2007). Left–right asymmetries in antennal sucrose processing may have, therefore, a neural correlate at the level of the lateral lobes with enhanced signaling on the right side compared to the left side. This possibility remains so far unexplored and further investigations will be necessary to understand the neural underpinnings of lateralization in the case of antennal sucrose responsiveness.

The adaptive value of the lateralized sucrose sensitivity at the level of the antennae remains unclear. From an ecological perspective, it would be interesting to determine whether foragers entering in contact with food, be it pollen or nectar, exhibit some bias prioritizing a first contact with the right antenna. As the right antenna is particularly sensitive to low sucrose concentrations (e.g., 3%), which correspond to those found in various pollen types (Szczęsna, 2007), pollen contact may be lateralized similarly to what occurs at the level of social interactions. In this case, bees display a lateral preference to use their right antenna in positive interactions with other bees involving food exchange (Rogers et al., 2013). Moreover, an antennal bias in sugar responsiveness could help optimizing side-specific odor memory formations and retention during foraging. Specializing the right side for immediate-short-term odor memory has been hypothesized to be of aid for building odor memories in a more efficient way to reduce interference of two types of neural processing (learning and recalling) during foraging activity over time (Rogers and Vallortigara, 2008). When bees are trained in the lab with both antennae in use, the recall of odor memories shows a shift of antenna dominance over time, with the right antenna specialized for short-term memory recall and the left one for long-term memory recall (Rogers and Vallortigara, 2008; Frasnelli et al., 2010b). Having the left antenna less tuned to sugar responsiveness could contribute to this specialization, leaving the left side free to perform a parallel task possibly tuned to long-term memory formation.

Similarly to what has been reported by previous works (Marshall, 1935; de Brito Sanchez et al., 2008), we found that the fore-tarsi were less sensitive to sucrose than the antennae, a fact that may be related to the different number of taste sensilla located on these gustatory structures [antennae count about 15–30 times more receptors than the tarsi (Whitehead and Larsen, 1976; de Brito Sanchez, 2011)]. Contrary to the case of the antennae, no evidence for lateralization of sucrose sensitivity was found at the level of the distal segments of the fore-tarsi. Each tarsomere has two types of gustatory sensilla, 10–21 *sensilla chaetica* and 0–6 *sensilla basiconica* (Whitehead and Larsen, 1976) but whether these numbers differ between the left and right fore-tarsi remains to be determined. Similarly, whether projections of the gustatory receptors hosted by these sensilla to the central level (i.e., to the thoracic ganglion and eventually to the SEZ) differ between sides is unknown. Our behavioral results do not seem to support the existence of differences in the number and/or sensitivity of gustatory receptors at either the peripheral or the central level.

An interesting finding concerning tarsal sucrose sensitivity was the significant effect of sequence stimulation. When bees were stimulated with increasing sucrose concentrations on one tarsus, sucrose sensitivity was increased in the contralateral tarsus, irrespective of the side considered. This result indicates that excitation induced by sucrose stimulations is transferred via central integration to the contralateral tarsus. Previous behavioral experiments showed that sucrose solution delivered on one tarsus elicits immediate PER which cannot be inhibited by any other aversive substance delivered afterward on the contralateral tarsus. In these conditions, sucrose was suggested to act as a “winner takes-all” stimulus, suggesting “a process of central integration, probably at the level of the thoracic ganglion” (de Brito Sanchez et al., 2014). This hypothesis is consistent with the present findings and indicates that when bees detect sucrose with one fore-tarsus, they become “prepared” to sense sucrose with the opposite tarsus, a mechanism that may serve efficient location of minute nectar sources. Importantly, this effect cannot be attributed to sensitization, the enhancement of responsiveness due to non-associative experience with a repeated biologically relevant stimulus like food (Squire and Kandel, 1999). In the honeybee, sensitization is only observable after very short intervals (seconds to few minutes) following food stimulation (Menzel, 1999). The fact that an interval of 2 h was interspersed between the two sucrose stimulation phases excludes the possibility of bees being sensitized by the first stimulation phase. Moreover, given the fact that the antennae are more sensitive to sucrose than the tarsi (see above), if sensitization would have occurred, it should have been observed at the level of the antennae rather than at the level of the tarsi. This was not the case and rules out, therefore, the possibility of sensitization accounting for enhanced responsiveness between fore-tarsi.

To date, clear anatomical asymmetries at the level of the brain are still lacking for honeybees (Haase et al., 2011a,b; Rigosi et al., 2011) and differences in the number or sensitivity of olfactory and non-olfactory sensilla are unlikely to explain entirely the behavioral laterality found in this insect (Frasnelli et al., 2012). Phenomena such as the lateral shift (Rogers and Vallortigara, 2008) or the side specificity of olfactory learning and generalization (Sandoz and Menzel, 2001; Sandoz et al., 2002) together with evidence of asymmetry in gene expression (Biswas et al., 2010; McNeill and Robinson, 2015; Guo et al., 2016; McNeill et al., 2016) and odor processing (Rigosi et al., 2015) described in honeybees suggest, indeed, that asymmetries at the central level also exist and await for better characterizations.

## Author Contributions

ER, GdBS, and MG conceived the study. DB designed the experiments, performed the experiments with the help of students, and carried out the data analysis. All authors contributed equally to the writing of the manuscript.

## Conflict of Interest Statement

The authors declare that the research was conducted in the absence of any commercial or financial relationships that could be construed as a potential conflict of interest.

## References

[B1] Anfora G. Frasnelli E. Maccagnani B. Rogers L. J. Vallortigara G. (2010). Behavioural and electrophysiological lateralization in a social (*Apis mellifera*) but not in a non-social (*Osmia cornuta*) species of bee. *Behav. Brain Res.* 206 236–239. 10.1016/j.bbr.2009.09.023 19766143

[B2] Baracchi D. Devaud J. M. d’Ettorre P. Giurfa M. (2017). Pheromones modulate reward responsiveness and non-associative learning in honey bees. *Sci. Rep.* 7:9875. 10.1038/s41598-017-10113-7 28852036 PMC5574997

[B3] Bates D. Mächler M. Bolker B. Walker S. (2014). Fitting linear mixed-effects models using lme4. arxiv.org/abs/1406.5823

[B4] Biswas S. Reinhard J. Oakeshott J. Russell R. Srinivasan M. V. Claudianos C. (2010). Sensory regulation of neuroligins and neurexin I in the honeybee brain. *PLoS One* 5:e9133. 10.1371/journal.pone.0009133 20161754 PMC2817746

[B5] Corballis M. C. (1989). Laterality and human evolution. *Psychol. Rev.* 96 492–505. 10.1037/0033-295X.96.3.492 2667014

[B6] de Brito Sanchez M. G. (2011). Taste perception in honey bees. *Chem. Senses* 36 675–692. 10.1093/chemse/bjr040 21622601

[B7] de Brito Sanchez M. G. Chen C. Li J. Liu F. Gauthier M. Giurfa M. (2008). Behavioral studies on tarsal gustation in honeybees: sucrose responsiveness and sucrose-mediated olfactory conditioning. *J. Comp. Physiol. A* 194 861–869. 10.1007/s00359-008-0357-8 18704443

[B8] de Brito Sanchez M. G. Giurfa M. de Paula Mota T. R. Gauthier M. (2005). Electrophysiological and behavioural characterization of gustatory responses to antennal ‘bitter’ taste in honeybees. *Eur. J. Neurosci.* 22 3161–3170. 10.1111/j.1460-9568.2005.04516.x 16367782

[B9] de Brito Sanchez M. G. Lorenzo E. Su S. Fanglin L. Zhan Y. Giurfa M. (2014). The tarsal taste of honey bees: behavioral and electrophysiological analyses. *Front. Behav. Neurosci.* 8:25. 10.3389/fnbeh.2014.00025 24550801 PMC3913880

[B10] Esslen J. Kaissling K. E. (1976). Zahl und verteilung antennaler sensillen bei der honigbiene (*Apis mellifera* L.). *Zoomorphology* 83 227–251. 10.1007/BF00993511

[B11] Frasnelli E. (2017). Brain and behavioral lateralization in invertebrates. *Front. Psychol.* 4:939. 10.3389/fpsyg.2013.00939 24376433 PMC3859130

[B12] Frasnelli E. Anfora G. Trona F. Tessarolo F. Vallortigara G. (2010a). Morpho-functional asymmetry of the olfactory receptors of the honeybee (*Apis mellifera*). *Behav. Brain Res.* 209 221–225. 10.1016/j.bbr.2010.01.046 20138089

[B13] Frasnelli E. Haase A. Rigosi E. Anfora G. Rogers L. J. Vallortigara G. (2014). The bee as a model to investigate brain and behavioural asymmetries. *Insects* 5 120–138. 10.3390/insects5010120 26462583 PMC4592634

[B14] Frasnelli E. Vallortigara G. Rogers L. J. (2010b). Response competition associated with right–left antennal asymmetries of new and old olfactory memory traces in honeybees. *Behav. Brain Res.* 209 36–41. 10.1016/j.bbr.2010.01.014 20085786

[B15] Frasnelli E. Vallortigara G. Rogers L. J. (2012). Left–right asymmetries of behaviour and nervous system in invertebrates. *Neurosci. Biobehav. Rev.* 36 1273–1291. 10.1016/j.neubiorev.2012.02.006 22353424

[B16] Guiraud M. Hotier L. Giurfa M. de Brito Sanchez M. G. (2018). Aversive gustatory learning and perception in honey bees. *Sci. Rep.* 8:1343. 10.1038/s41598-018-19715-1 29358592 PMC5778057

[B17] Guo Y. Wang Z. Li Y. Wei G. Yuan J. Sun Y. (2016). Lateralization of gene expression in the honeybee brain during olfactory learning. *Sci. Rep.* 6:34727. 10.1038/srep34727 27703214 PMC5050455

[B18] Haase A. Rigosi E. Frasnelli E. Trona F. Tessarolo F. Vinegoni C. (2011a). A multimodal approach for tracing lateralisation along the olfactory pathway in the honeybee through electrophysiological recordings, morpho-functional imaging, and behavioural studies. *Eur. Biophys. J.* 40 1247–1258. 10.1007/s00249-011-0748-6 21956452 PMC3366498

[B19] Haase A. Rigosi E. Trona F. Anfora G. Vallortigara G. Antolini R. (2011b). In-vivo two-photon imaging of the honey bee antennal lobe. *Biomed. Opt. Express* 2 131–138. 10.1364/BOE.1.000131 21326643 PMC3028488

[B20] Haupt S. S. (2004). Antennal sucrose perception in the honey bee (*Apis mellifera* L.): behaviour and electrophysiology. *J. Comp. Physiol. A* 190 735–745. 10.1007/s00359-004-0532-5 15300385

[B21] Haupt S. S. (2007). Central gustatory projections and side-specificity of operant antennal muscle conditioning in the honeybee. *J. Comp. Physiol. A* 193 523–535. 10.1007/s00359-007-0208-z 17265152

[B22] Letzkus P. Boeddeker N. Wood J. T. Zhang S. W. Srinivasan M. V. (2008). Lateralization of visual learning in the honeybee. *Biol. Lett.* 4 16–19. 10.1098/rsbl.2007.0466 18029300 PMC2412924

[B23] Letzkus P. Ribi W. A. Wood J. T. Zhu H. Zhang S. W. Srinivasan M. V. (2006). Lateralization of olfaction in the honeybee *Apis mellifera*. *Curr. Biol.* 16 1471–1476. 10.1016/j.cub.2006.05.060 16860748

[B24] Marshall J. (1935). On the sensitivity of the chemoreceptors on the antenna and fore-tarsus of the honey-bee, (*Apis mellifica* L.). *J. Exp. Biol.* 12 17–26.

[B25] Matsumoto Y. Menzel R. Sandoz J. C. Giurfa M. (2012). Revisiting olfactory classical conditioning of the proboscis extension response in honey bees: a step toward standardized procedures. *J. Neurosci. Methods* 211 159–167. 10.1016/j.jneumeth.2012.08.018 22960052

[B26] McNeill M. Kapheim K. Brockmann A. McGill T. Robinson G. (2016). Brain regions and molecular pathways responding to food reward type and value in honey bees. *Genes Brain Behav.* 15 305–317. 10.1111/gbb.12275 26566901

[B27] McNeill M. Robinson G. (2015). Voxel-based analysis of the immediate early gene, c-jun, in the honey bee brain after a sucrose stimulus. *Insect Mol. Biol.* 24 377–390. 10.1111/imb.12165 25773289

[B28] Menzel R. (1999). Memory dynamics in the honeybee. *J. Comp. Physiol. A* 185 323–340. 10.1007/s003590050392

[B29] Pankiw T. Page R. Jr. (1999). The effect of genotype, age, sex, and caste on response thresholds to sucrose and foraging behavior of honey bees (*Apis mellifera* L.). *J. Comp. Physiol. A* 185 207–213. 10.1007/s003590050379 10488557

[B30] Pareto A. (1972). Die zentrale verteilung der fühlerafferenz bei arbeiterinnen der honigbiene, *Apis mellifera* L. *Cell Tissue Res.* 131 109–140. 5073638

[B31] R Core Team (2016). *R: A Language and Environment for Statistical Computing*. Vienna: R Foundation for Statistical Computing.

[B32] Rigosi E. Frasnelli E. Vinegoni C. Antolini R. Anfora G. Vallortigara G. (2011). Searching for anatomical correlates of olfactory lateralization in the honeybee antennal lobes: a morphological and behavioural study. *Behav. Brain Res.* 221 290–294. 10.1016/j.bbr.2011.03.015 21402106 PMC3089663

[B33] Rigosi E. Haase A. Rath L. Anfora G. Vallortigara G. Szyszka P. (2015). Asymmetric neural coding revealed by in vivo calcium imaging in the honey bee brain. *Proc. Biol. Sci. B* 282:20142571. 10.1098/rspb.2014.2571 25673679 PMC4345443

[B34] Rogers L. J. Vallortigara G. (2008). From antenna to antenna: lateral shift of olfactory memory recall by honeybees. *PLoS One* 3:e2340. 10.1371/journal.pone.0002340 18523636 PMC2394662

[B35] Rogers L. J. Vallortigara G. (2017). *Lateralized Brain Functions: Methods in Human and Non-Human Species*. Berlin: Springer 10.1007/978-1-4939-6725-4

[B36] Rogers L. J. Vallortigara G. Andrew R. J. (2013). *Divided Brains: The Biology and Behaviour of Brain Asymmetries*. Cambridge: Cambridge University Press 10.1017/CBO9780511793899

[B37] Sandoz J. C. Hammer M. Menzel R. (2002). Side-specificity of olfactory learning in the honeybee: US input side. *Learn. Mem.* 9 337–348. 10.1101/lm.50502 12359841 PMC187131

[B38] Sandoz J. C. Menzel R. (2001). Side-specificity of olfactory learning in the honeybee: generalization between odors and sides. *Learn. Mem.* 8 286–294. 10.1101/lm.41401 11584076 PMC311384

[B39] Scheiner R. (2004). Responsiveness to sucrose and habituation of the proboscis extension response in honey bees. *J. Comp. Physiol. A* 190 727–733. 10.1007/s00359-004-0531-6 15185117

[B40] Scheiner R. Barnert M. Erber J. (2003). Variation in water and sucrose responsiveness during the foraging season affects proboscis extension learning in honey bees. *Apidologie* 34 67–72. 10.1051/apido:2002050

[B41] Scheiner R. Erber J. Page R. Jr. (1999). Tactile learning and the individual evaluation of the reward in honey bees (*Apis mellifera* L.). *J. Comp. Physiol. A Neuroethol. Sens. Neural Behav. Physiol.* 185 1–10. 10.1007/s003590050360 10450609

[B42] Scheiner R. Page R. E. Erber J. (2001). Responsiveness to sucrose affects tactile and olfactory learning in preforaging honey bees of two genetic strains. *Behav. Brain Res.* 120 67–73. 10.1016/S0166-4328(00)00359-4 11173086

[B43] Schröter U. Malun D. Menzel R. (2006). Innervation pattern of suboesophageal VUM neurons in the honeybee brain. *Cell Tissue Res.* 326 647–667. 17093927 10.1007/s00441-006-0197-1

[B44] Squire L. R. Kandel E. R. (1999). *Memory: From Mind to Molecules. New York: Scientific American Library*. New York, NY: W.H. Freeman.

[B45] Suzuki H. (1975). Antennal movements induced by odour and central projection of the antennal neurones in the honey-bee. *J. Insect Physiol.* 21 831–847. 10.1016/0022-1910(75)90012-8

[B46] Suzuki H. Thiele T. R. Faumont S. Ezcurra M. Lockery S. R. Schafer W. R. (2008). Functional asymmetry in *Caenorhabditis elegans* taste neurons and its computational role in chemotaxis. *Nature* 454 114–117. 10.1038/nature06927 18596810 PMC2984562

[B47] Szczęesna T. (2007). Study on the sugar composition of honeybee-collected pollen. *J. Apic. Sci.* 51 15–22.

[B48] Takeda K. (1961). Classical conditioned response in the honey bee. *J. Insect Physiol.* 6 168–179. 10.1016/0022-1910(61)90060-9

[B49] Vallortigara G. Rogers L. J. (2005). Survival with an asymmetrical brain: advantages and disadvantages of cerebral lateralization. *Behav. Brain Sci.* 28 575–588. 10.1017/S0140525X05000105 16209828

[B50] Wes P. D. Bargmann C. I. (2001). *C. elegans* odour discrimination requires asymmetric diversity in olfactory neurons. *Nature* 410 698–701. 10.1038/35070581 11287957

[B51] Whitehead A. Larsen J. (1976). Ultrastructure of the contact chemoreceptors of *Apis mellifera* L. (Hymenoptera: Apidae). *Int. J. Insect Morphol. Embryol.* 5 301–315. 10.1016/0020-7322(76)90030-1

